# Body composition and functional capacity as determinants of physical activity in middle-aged and older adults: a cross-sectional analysis

**DOI:** 10.1186/s11556-025-00372-z

**Published:** 2025-05-01

**Authors:** Matti Hyvärinen, Anna Kankaanpää, Timo Rantalainen, Taina Rantanen, Eija K. Laakkonen, Laura Karavirta

**Affiliations:** Gerontology Research Center, Faculty of Sport and Health Sciences, University of Jyväskylä, Jyväskylä, Finland

**Keywords:** Physical performance, Physical fitness, Muscle mass, Fat mass, Obesity, Exercise

## Abstract

**Background:**

Body composition and functional capacity are both related to physical activity, but the interplay is complex, as different body tissue types contribute differently on physical activity and functional capacity. To clarify the role of body composition and functional capacity as determinants of physical activity in aging, we investigated the associations of different body tissue types, muscle strength, and walking capacity with physical activity in middle-aged and older adults.

**Methods:**

This cross-sectional study involved 1158 women aged 47–55 years, and community-dwelling 289 women and 196 men aged 75, 80, or 85 years. Their physical activity was assessed with accelerometers, muscle mass and muscle-free mass with bioelectrical impedance analysis, walking performance with a six-minute walking test, and muscle strength with maximal isometric knee extension test. The associations of muscle mass, muscle-free mass, walking performance, and muscle strength with physical activity were studied separately for middle-aged women, older women, and older men using linear regression and structural equation models.

**Results:**

Total body mass and absolute muscle-free mass were inversely associated with the level of physical activity in all study groups. Furthermore, walking performance, muscle strength, and muscle mass relative to body weight, but not absolute muscle mass, were directly associated with the level of physical activity. The associations between the measures of body composition and physical activity were fully explained by the differences in functional capacity, as defined by both walking performance and muscle strength, in structural equation models. Functional capacity was strongly associated with higher levels of physical activity regardless of body composition, especially among older people: β = 0.70, SE = 0.10 for older women, β = 0.92, SE = 0.27 for older men, and β = 0.41, SE = 0.10 for middle-aged women.

**Conclusions:**

Reduced functional capacity may be a key factor limiting physical activity, with its impact becoming more pronounced as functional capacity declines with age. Based on these findings, maintaining and improving functional capacity is essential for promoting an active lifestyle in older adults.

**Supplementary Information:**

The online version contains supplementary material available at 10.1186/s11556-025-00372-z.

## Introduction

Regular physical activity improves the quality of life and is beneficial for the prevention and management of several chronic diseases and other public health concerns, such as cardiovascular disease, diabetes, cancer, hypertension, and osteoporosis, which are especially common in older adults [1, 2]. Furthermore, physical inactivity, defined as failing to meet the minimum recommendations for physical activity, is the fourth leading cause of death worldwide [3]. Despite the well-known benefits of physical activity, less than 45% of older adults are sufficiently active to maintain their health, and the prevalence of physical inactivity is increasing, especially in high-income countries [4, 5]. This highlights the need to study the underlying factors of physical behaviour to provide tools for health promotion in middle-aged and older adults.

Previous studies have identified several potential determinants of physical activity in adults, including genotype, sex, gender, ethnicity, and various modifiable traits, such as functional capacity and obesity [6, 7]. Moreover, in our study of older adults, we found that differences in physical activity levels between sexes were primarily accounted by differences in body composition and functional capacity, specifically body adiposity and cardiorespiratory fitness [8]. However, the interplay between body composition, functional capacity, and physical activity is complex. Greater body weight and adiposity increase the force and energy demands of the bodily movement causing reduction in functional capacity [9–11]. Consequently, obese individuals tend to be less active compared to individuals with normal weight [7]. Since physical activity has only a minor role in weight management, it is more likely that the inverse associations between body weight and physical activity results from the effect of body weight on physical activity than vice versa [12]. However, all tissues of the body are not the same in terms of the functional capacity. Particularly, greater skeletal muscle mass associates with increased muscle strength and improved functional capacity [13, 14]. Thus, the lack of muscle mass may have a significant negative influence on the ability to engage in physical activity, especially in older adults [15].

Despite the complex interplay between body composition, functional capacity, and physical activity, previous studies have not comprehensively explored the role of different body tissue types as a determinant of physical activity. Therefore, the aim of this study was to conduct exploratory cross-sectional analyses on the associations between muscle mass, muscle-free mass, functional capacity, and physical activity in two separate cohorts of middle-aged women and older adults. These cohorts provide a unique opportunity to study the role of body composition and functional capacity as determinants of physical activity since the menopausal transition in women and old age in both men and women are phases in life that are characterised by the increases in body adiposity as well as decreases in skeletal muscle mass and functional capacity [16–19]. Based on the known role of body adiposity and skeletal muscle mass on functional capacity, we hypothesize that greater skeletal muscle mass and lower total body and muscle-free mass are associated with higher physical activity levels, and that this relationship is primarily explained by differences in functional capacity, which describes the ability to move one’s body mass.

## Methods

### Participants and study design

This study was conducted using baseline data from two separate population-based cohort studies that were (1) The Estrogenic Regulation of Muscle Apoptosis (ERMA) and (2) Active Aging– Resilience and External Support as Modifiers of the Disablement Outcome (AGNES). The participant selection procedures and study protocols for both ERMA [20] and AGNES [21] studies have been reported in detail previously.

The ERMA study was designed to study the role of menopausal transition in physiological and psychological functioning in middle-aged women [20]. A random sample of 6 878 women aged 47–55 living in the city of Jyväskylä and neighbouring municipalities in Central Finland drawn from the Digital and Population Data Services Agency (DPDSA) population register were invited to participate in the baseline measurements. Of those women, 3 649 did not respond to the invitation, 421 were not willing to participate, 445 did not consent, and 1 205 were excluded. The exclusion criteria of the study included conditions affecting systemic hormone levels, inflammatory status, or ovarian function, such as bilateral oophorectomy, lactation, pregnancy, and severe obesity with self-reported body mass index (BMI) greater than 35 kg/m^2^. Consequently, 1 158 women participated in the ERMA baseline laboratory visits with physiological measurements. These measurements were carried out in 2015–2016.

The AGNES study targeted 75-, 80-, and 85-year-old people living independently in the city of Jyväskylä, in Central Finland [21]. The invitation to participate was sent to a random sample of 2 791 older men and women drawn from the DPDSA population register. Of the invited older men and women, 434 did not respond, 1 218 were not willing to participate or did not consent, and 118 were excluded [22, 23]. The participants were excluded if they did not live independently in the recruitment area or were unable to provide meaningful responses. Consequently, 1 021 older men and women participated in the AGNES baseline measurements, of which 485 participants (196 men, 289 women) agreed to participate and provided a valid accelerometer-based physical activity recording. The baseline measurements were carried out in 2017–2018.

Both studies were approved by the Ethical Committee of the Central Finland Health Care District (ERMA DN 8U/2014 and AGNES DN 14U/2017) and all participants provided written informed consent before the assessments. The studies were performed in accordance with the Declaration of Helsinki.

### Physical activity

Physical activity was assessed with mean amplitude deviations (MAD) of tri-axial accelerometer data in both studies. In the ERMA study, participants wore accelerometers (ActiGraph GT3X or wGT3X, ActiGraph, Pensacola, FL) on their hip for seven consecutive days during waking hours. The data were collected at the frequency of 60 Hz. The AGNES study utilized thigh-worn accelerometers (UKK RM42, UKK Terveyspalvelut Oy, Tampere, Finland) suitable for continuous wear. Participants were instructed to wear the accelerometer for a minimum of seven consecutive days and the data were collected using the frequency of 100 Hz. In both studies, MAD values were computed for non-overlapping five-second epochs based on the Euclidian norm of the resultant acceleration. Physical activity was assessed as the mean of all MAD values. A wear time of 10 h or more in ERMA and 24 h in AGNES for a minimum of three days was regarded as a valid measurement. The raw accelerometer data analyses conducted in ERMA [24] and AGNES [25, 26] studies have been reported in detail elsewhere.

### Body composition and functional capacity

Skeletal muscle mass and total body mass were assessed with bioelectrical impedance analysis (InBody720, Biospace, Seoul, Korea) according to the manufacturer’s instructions. The ERMA measurements were conducted in the morning after overnight fasting [20]. In the AGNES study, participants were instructed to consume only a light meal before arriving to the research centre on the measurement day [21]. The relative skeletal muscle mass was computed by dividing the skeletal muscle mass by total body mass, and muscle-free mass was determined as the difference between total body mass and skeletal muscle mass.

Functional capacity was measured by assessing lower extremity muscle strength with maximal isometric knee extension test and cardiorespiratory fitness with six-minute walk test. In both studies, the maximal isometric knee extension force of the dominant leg was measured at a knee angle of 60 degrees from the full extension using an adjustable dynamometer chair (Metitur Ltd, Jyväskylä, Finland) [20, 21]. For each participant, the test was performed at least three times, with a one-minute rest between trials, until no further improvement was observed. The maximal knee extension torque in Newton metres was computed by multiplying the maximal knee extension force by the individual lever arm used in the test. The maximal knee extension torque relative to total body mass was used in the analyses [27].

The six-minute walk test was performed on the track set up in the indoor corridor by placing traffic cones 20 m apart, resulting in a 40-meter lap. In the ERMA study, participants were instructed to walk at the maximal walking speed in order to complete as many laps as possible during the test [20]. In the AGNES study, the six-minute walk test was modified for better fit for older participants. Consequently, the AGNES participants were instructed to use their preferred rather than maximal walking speed to ensure safety and to promote continuous walking performance during the whole six-minute period [21]. The total distance walked during the test was measured.

### Covariates

Since ERMA participants were menopausal and most of them were involved in working life, unlike the AGNES participants, menopausal status and type of the work (occupational physical activity) were assessed for the ERMA participants. In the menopausal status assignments, the participants were categorized as pre-, peri-, or postmenopausal based on the follicle-stimulating hormone concentrations and self-reported menstrual bleeding diaries using the adapted Stages of Reproductive Aging Workshop (STRAW + 10) guidelines [20, 28]. The follicle-stimulating hormone concentrations were determined with immunoassays (IMMULITE 2000 XPi, Siemens Healthineers, Erlangen, Germany) from the fasting blood samples taken during the first five days of the menstrual cycle for participants with predictable cycles. Additionally, the participants provided a diary that included information about the menstrual bleedings during the six-month period prior to the measurements. Occupational physical activity was assessed using a structured question [20]. Based on the responses, participant’s work type was classified as either sedentary work or work that includes physical activity.

### Statistical analyses

The associations between measures of physical activity, body composition, and functional capacity were studied using linear regression models and Pearson correlation coefficients. In linear regression models, physical activity was used as an outcome variable and standardized regression coefficients were reported. Residual plots, Q–Q plots, correlation coefficients, and variance inflation factors were studied to ensure that there were no significant violations of the model assumptions. The analyses were carried out in R (version 4.3.3) [29].

For the analysis of indirect associations, we used structural equation models to study the role of muscle mass, muscle-free mass, and functional capacity as determinants of physical activity. Both muscle mass and muscle-free mass were included in the models because we considered them key components of body composition, given their distinct effects on functional capacity. In the models, functional capacity was a latent variable defined by the six-minute walking distance and relative knee extension torque and it was treated as an intervening variable. The indirect effects of muscle mass and muscle-free mass on physical activity through functional capacity were calculated as products of the path coefficients. The model parameters were estimated using the full information maximum likelihood method with robust standard errors (FIML). The goodness-of-fit for the models was assessed using the Satorra-Bentler corrected χ^2^-test, comparative fit index (CFI), Tucker-Lewis index (TLI), the root mean square error of approximation (RMSEA), and standard root mean residual (SRMR). The model fits the data well if the *p*-value for the χ^2^-test is greater than 0.05, CFI and TLI are close to 0.95, and RMSEA and SRMR are less than 0.08 [30]. The strength of the association is interpreted using the standardized coefficients, with threshold values of 0.1, 0.3, and 0.5 representing weak, moderate, and strong associations, respectively [31]. The models were conducted using the Mplus software (version 8.4) [32].

All models were constructed separately for the ERMA study, AGNES women, and AGNES men. The models for the AGNES participants were constructed separately for men and women to facilitate the interpretation and comparison of the results between the studies since ERMA study included only women. All structural equation models and multiple linear regression models were adjusted for age. Additionally, the models for the ERMA participants were adjusted for occupational physical activity and menopausal status.

### Missing data

For the ERMA participants, the number of missing data values was 2 115 out of 12 738 (17%) and the percentage of missing data values varied from 0 to 32% across the variables. For the AGNES participants, there were 169 missing data values out of 4 850 (3%) and the percentage of missing data values varied from 0 to 7% across the variables. Missing data occurred due to invalid or missing measurements and unclear and incomplete questionnaire responses. Missing data were assumed to occur at random (MAR) [33]. Multiple imputation was not used for missing physical activity data in the AGNES study because one of the most reported reasons for declining participation in the accelerometer-based assessment was a lack of physical activity. Thus, the missingness was not random.

For linear regression models, multiple imputation with 50 imputed data sets and 50 iterations for chained equations was used to deal with the missing data. Passive imputation was used for the imputation of derived variables. All available variables other than the variable to impute were used for the imputation, except for the derived variables that were not used for the imputation of their originals. Multiple imputation and pooling of the regression coefficients were carried out in R using the standard settings of the “mice” package [34]. We also conducted complete case analyses, and the results did not differ notably from the ones acquired using multiple imputation. Multiple imputation was not used for the structural equation models because the FIML estimator used in the analyses provides unbiased estimates if the mechanism of missingness is MAR [35].

## Results

### Characteristics of the study populations

The mean age of middle-aged women in the ERMA study was 51.4 years, while the mean ages for older women and men in the AGNES study were 78.3 and 78.4 years, respectively (Table 1). On average, the participants were slightly overweight with the mean BMI of 25.5, 28.0, and 26.8 kg/m^2^ respectively for middle-aged women, older women, and older men. The corresponding body fat percentages were 30.9%, 39.1%, and 27.7%.

**Table 1:** Characteristics of the study populations

	Middle-aged women	Older women	Older men
**Background characteristics**	*n* = 1158	*n* = 289	*n* = 196
Age [year]	51.4 ± 2.0	78.3 ± 3.5	78.4 ± 3.4
Menopausal status^a^			
Pre	27.2 (315)		
Peri	37.3 432)	n.a.	n.a.
Post	35.5 (411)		
Occupational physical activity^a^			
Sedentary work	46.2 (535)	n.a.	n.a.
Includes physical activity	40.9 (474)		
**Physical activity**	*n* = 784	*n* = 289	*n* = 196
Mean MAD [mg]	29.2 ± 9.2	23.0 ± 8.0	25.8 ± 8.6
**Anthropometrics and body composition**	*n* = 1018	*n* = 279	*n* = 179
Body height [cm]	165 ± 6	158 ± 5	172 ± 6
Total body mass [kg]	70.0 ± 11.0	70.3 ± 12.2	79.7 ± 11.5
Body mass index [kg/m^2^]	25.5 ± 3.7	28.0 ± 4.8	26.8 ± 3.7
Body fat percentage [%]	30.9 ± 7.5	39.1 ± 7.4	27.7 ± 7.1
Muscle-free mass [kg]	43.6 ± 9.1	47.8 ± 10.5	48.3 ± 9.1
Muscle mass [kg]	26.4 ± 3.1	22.5 ± 2.7	31.4 ± 4.0
Relative muscle mass [%]	38.2 ± 4.2	32.5 ± 4.0	39.7 ± 4.0
**Functional capacity**			
Six-minute walking distance [m]	669 ± 61 (*n* = 850)	408 ± 79 (*n* = 279)	435 ± 81 (*n* = 189)
Relative knee extension torque [Nm/kg]	2.28 ± 0.51 (*n* = 794)	1.05 ± 0.32 (*n* = 275)	1.60 ± 0.40 (*n* = 175)

Data are mean ± standard deviation unless otherwise specified. ^a^ Data are % (n); MAD; mean amplitude deviation; mg, milligravity (0.00981 m/s^2^)

Among the three separate study groups, middle-aged women walked longer distance during the six-minute walking test compared to older men and women (Table 1). However, due to the different study protocols used in the six-minute walk test, the results are not fully comparable between middle-aged women and older adults. For older adults, men were physically more active and walked longer distance during the six-minute walking test compared to women. Furthermore, middle-aged women had the greatest maximal knee extension torque relative to total body mass and older men had greater relative knee extension torque compared to older women. Middle-aged women and older men had more muscle mass relative to total body mass (38 and 40%, respectively) compared to older women (33%).

### Associations of body composition and functional capacity with physical activity

Total body mass and muscle-free mass were inversely associated with physical activity and these inverse associations tended to be stronger in older women compared to other groups (Table 2). Absolute muscle mass was not associated with physical activity in any of the study groups. However, muscle mass relative to total body mass and the measures of functional capacity had moderate positive associations with physical activity in all groups. These associations tended to be stronger in older adults (β = 0.33–0.51) compared to middle-aged women (β = 0.22–0.28). Muscle-free mass explained most of the variance in total body mass in all groups with Pearson correlation coefficient varying from 0.95 to 0.97 between muscle-free mass and total body mass (Additional Table 1). The confounders did not substantially affect the associations of body composition and functional capacity with physical activity.

**Table 2:** Associations of body composition and the measures of functional capacity with physical activity

	β	95% CI
**Middle-aged women (*n*** **= 1158)**		
Total body mass [kg] ^a^	-0.175 ***	[-0.243, -0.107]
Muscle-free mass [kg] ^a^	-0.218 ***	[-0.285, -0.152]
Muscle mass [kg] ^a^	0.021	[-0.050, 0.093]
Relative muscle mass [%] ^a^	0.273 ***	[0.205, 0.340]
Six-minute walking distance [m] ^a^	0.284 ***	[0.217, 0.350]
Relative knee extension torque [Nm/kg] ^a^	0.220 ***	[0.149, 0.292]
**Older women (*n*** **= 289)**		
Total body mass [kg]	-0.322 ***	[-0.431, -0.213]
Muscle-free mass [kg]	-0.364 ***	[-0.472, -0.258]
Muscle mass [kg]	-0.030	[-0.149, 0.087]
Relative muscle mass [%]	0.405 ***	[0.298, 0.512]
Six-minute walking distance [m]	0.510 ***	[0.401, 0.619]
Relative knee extension torque [Nm/kg]	0.444 ***	[0.337, 0.550]
**Older men (*n*** **= 196)**		
Total body mass [kg]	-0.195 **	[-0.337, -0.052]
Muscle-free mass [kg]	-0.285 ***	[-0.425, -0.145]
Muscle mass [kg]	0.115	[-0.037, 0.267]
Relative muscle mass [%]	0.410***	[0.266, 0.554]
Six-minute walking distance [m]	0.410***	[0.266, 0.554]
Relative knee extension torque [Nm/kg]	0.328***	[0.188, 0.469]

Multiple imputation was applied in the analyses. The outcome in the models is physical activity as mean amplitude deviation [mg]. All models are adjusted for age. ^a^ Model is additionally adjusted for menopausal status and occupational physical activity; β, standardized regression coefficient; CI, confidence interval. *** *p* < 0.001, ** *p* < 0.01, * *p* < 0.05

### Muscle mass and functional capacity as determinants of physical activity

**Fig. 1:**
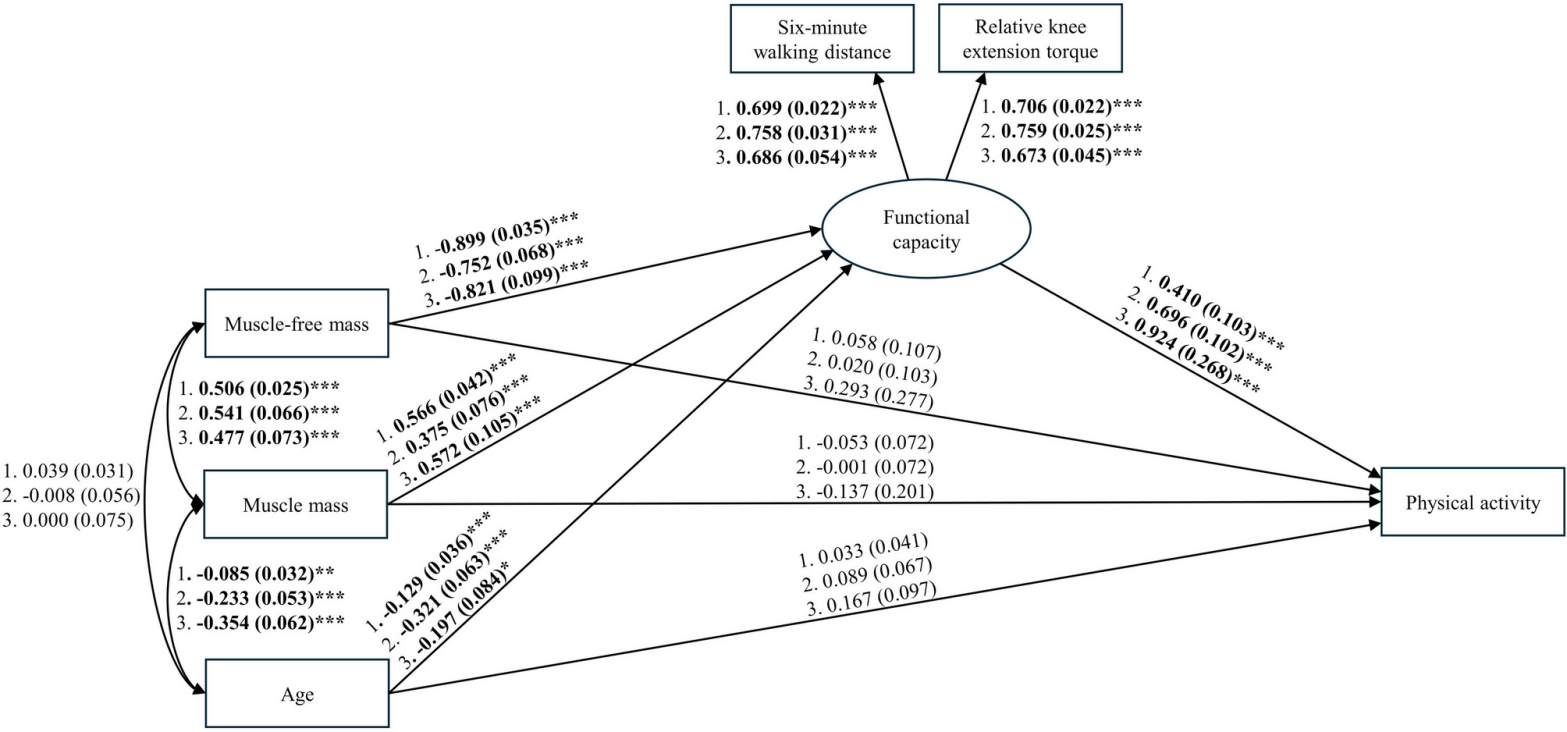
Structural equation models for all study groups with standardized path coefficients (standard error). Legend: Models were conducted separately for **1**) Middle-aged women (*n* = 1 158), **2**) Older women (*n* = 289), and **3**) Older men (*n* = 189). Model for middle-aged women is adjusted for menopausal status and occupational physical activity. *** *p* < 0.001, ** *p* < 0.01, * *p* < 0.05

In the structural equation models, neither muscle-free mass nor muscle mass were directly associated with physical activity (Table 3). However, muscle-free mass had strong inverse association with the latent variable of functional capacity in all groups with standardized path coefficients varying from − 0.90 to -0.75 (Fig. 1). Furthermore, greater muscle mass was moderately associated with functional capacity (β = 0.38–0.57). Functional capacity had strong positive association with physical activity in all study groups, and the associations were stronger in older men (β = 0.92) and women (β = 0.70) compared to middle-aged women (β = 0.41). Accordingly, both lower muscle-free mass and greater muscle mass were associated with greater physical activity indirectly via functional capacity (Table 3). Older age was associated with lower functional capacity and muscle mass in all study groups, but it was not directly associated with muscle-free mass nor physical activity (Fig. 1). Full structural equation models with standardized path coefficients and 95% confidence intervals are presented in Additional Tables 2, 3 and 4.

The model fit indices for structural equations model for middle-aged women were χ^2^ = 10.705, df = 7, *p* = 0.152, CFI = 0.995, TLI = 0.985, RMSEA = 0.021, and SRMR = 0.012. Respectively, the fit indices for older women were χ^2^ = 10.210, df = 4, *p* = 0.037, CFI = 0.981, TLI = 0.943, RMSEA = 0.073, SRMR = 0.020 and for older men χ^2^ = 9.141, df = 4, *p* = 0.058, CFI = 0.969, TLI = 0.906, RMSEA = 0.081, SRMR = 0.035. Thus, the constructed models fit well for the data of middle-aged and older men and satisfactorily for the data of older women.

**Table 3:** Direct and indirect associations of muscle-free mass and muscle mass on physical activity

	Direct associations		Indirect associations	
	β	95% CI	β	95% CI
**Middle-aged women (*n*** **= 1158)**^**a**^				
Muscle-free mass	0.058	[-0.151, 0.268]	-0.368 ***	[-0.562, -0.175]
Muscle mass	-0.053	[-0.193, 0.087]	0.232 ***	[0.108, 0.356]
**Older women (*n*** **= 289)**				
Muscle-free mass	0.020	[-0.183, 0.222]	-0.523 ***	[-0.722, -0.325]
Muscle mass	-0.001	[-0.142, 0.140]	0.261 ***	[0.131, 0.392]
**Older men (*n*** **= 196)**				
Muscle-free mass	0.293	[-0.250, 0.836]	-0.759 ***	[-1.315, -0.202]
Muscle mass	-0.137	[-0.530, 0.256]	0.528 ***	[0.149, 0.907]]

All models are adjusted for age. ^a^ Model is additionally adjusted for menopausal status and occupational physical activity; β, standardized path coefficient; CI, confidence interval. *** *p* < 0.001, ** *p* < 0.01, * *p* < 0.05

## Discussion

In this exploratory cross-sectional study, we observed that total body mass, body composition and the measures of functional capacity were associated with the level of physical activity in two separate cohorts of middle-aged women and older adults. However, the analysis of indirect effects indicated that body composition is not directly associated with the level of physical activity but its role as a determinant of physical activity is explained by the differences in functional capacity. Notably, greater functional capacity was strongly associated with higher levels of physical activity and these associations were stronger in older adults compared to middle-aged women.

Previous studies have reported that middle-aged and older adults with lower relative muscle mass and greater total body mass and higher adiposity levels tend to be less physically active [7, 36–38]. Interestingly, the relationship between body composition and physical activity is considered bidirectional, with exercise interventions being effective for reducing fat mass and increasing muscle mass, while body composition can influence individual’s ability to engage in physical activity [36, 39]. Our findings, which showed that lower total and muscle-free-mass, greater relative muscle mass, six-minute walking distance, and relative knee extension torque were weakly or moderately associated with higher levels of physical activity, agree with previous findings [38, 40]. However, our additional finding that absolute muscle mass was not associated with the level of physical activity suggests that the relationship between body composition and physical activity may be primarily driven by one’s capability to move their body weight.

In the analysis of indirect associations, we found out that the role of muscle-free mass and muscle mass as a determinant of physical activity is fully explained by the differences in functional capacity. More specifically, muscle-free mass and muscle mass had strong inverse and moderate direct associations with functional capacity, respectively. Functional capacity was a strong predictor of physical activity, and the associations were more evident with older adults compared to middle-aged women. These results are consistent with previous findings that better functional capacity is associated with higher level of physical activity, with associations becoming more pronounced with age [38, 41–43]. Our results indicate that previously reported association of total body mass and body composition with physical activity [7, 8] is mainly explained by the differences in functional capacity, which is a particularly strong determinant of physical activity. Furthermore, with the observed associations of functional capacity and physical activity being more pronounced in older adults compared to middle-aged women, our results suggest that the age-related decline in functional capacity may significantly contribute to the lack of physical activity in older adults.

Although our suggested models indicate that functional capacity is a strong determinant of physical activity, we cannot exclude the possibility of reverse causality in this cross-sectional study. The role of physical activity in maintaining better functional capacity in older adults is well-known, and several previous studies have reported that both resistance and aerobic training interventions are effective for improving physical functioning [44, 45]. However, it is unlikely that aerobic physical activity, assessed using accelerometers that detect movement rather than muscle activation, would have a significant impact on maximal knee extension torque, which was used as a measure of functional capacity together with six-minute walking distance in this study. Nonetheless, additional longitudinal and experimental studies with improved potential for causal inference are required to validate the role of functional capacity and its age-related changes as determinants of physical activity.

An interesting additional finding was that age was not directly linked with the level of physical activity in our models; however, it was inversely associated with muscle mass and functional capacity in all study groups, with stronger relationships observed in older adults compared to middle-aged women. These findings regarding muscle mass and functional capacity are supported by previous evidence on pronounced age-related decline in both functional capacity and muscle mass in older adults [46, 47]. Based on these findings, the well-known age-related decline in physical activity is primarily driven by the concurrent decline in functional capacity. Moreover, these results suggest that assessing functional capacity may provide a framework for the comparison of accelerometer-based physical activity metrics across different age groups, which would help clarify age-related differences in physical activity patterns and provide valuable insights for designing personalized physical activity recommendations.

A significant strength of this study is the use of the data from two relatively large cohort studies with accelerometer-based physical activity measurement and multiple measures of functional capacity, which enabled the possibility to investigate comprehensively the associations of interest with both middle-aged and older adults. The limitations of this study are the somewhat homogenous study samples of white men and women and the cross-sectional study design that limits the generalizability of the results to more heterogeneous populations and the ability to infer causality. Furthermore, differences in physical activity assessment and in the implementation of the six-minute walking test across cohorts as well as the lack of data from middle-aged men hinder the comparison of results between middle-aged and older adults in this study. Finally, the results of this study are susceptible to selection bias, particularly in the AGNES study, where less active participants were more likely to decline the physical activity assessment.

## Conclusion

This study provides preliminary evidence that functional capacity is a significant determinant of physical activity when assessed with an accelerometer, particularly in older adults. Furthermore, body composition does not appear to influence physical activity independently of functional capacity. These results suggest that reduced functional capacity may be a key factor limiting physical activity, with its impact becoming more pronounced as functional capacity declines with age. Based on these findings, adjusting accelerometer-based metrics of physical activity with functional capacity may enable comparison of physical behaviour across middle and older age. Furthermore, maintaining and improving functional capacity is essential for promoting an active lifestyle in older adults. However, further experimental and longitudinal studies are warranted to confirm the role of functional capacity as a determinant of physical activity.

## Electronic supplementary material

Below is the link to the electronic supplementary material.

Supplementary Material 1

## Data Availability

The datasets used in the study are not fully publicly available due to EU and Finnish legislation and the consent provided by the participants, which do not permit open access to individual level personal data. However, the metadata of the studies is published (ERMA: 10.17011/jyx/dataset/83491 and AGNES: 10.17011/jyx/dataset/83811) and the pseudonymized data are available on reasonable request to external collaborators upon agreement on the terms of data use and publication of results.

## Abbreviations

AGNES: Active aging– resilience and external support as modifiers of the disablement outcome
CFI: Comparative fit index
CI: Confidence interval
DPDSA: Digital and population data services agency
ERMA: Estrogenic regulation of muscle apoptosis
FIML: Full information maximum likelihood method with robust standard errors
MAD: Mean amplitude deviation
MAR: Missing at random
RMSEA: Root mean square error of approximation
SE: Standard error
SRMR: Standard root mean residual
TLI: Tucker-Lewis index
