# Supplementary material for: Body composition and functional capacity as determinants of physical activity in middle-aged and older adults: a cross-sectional analysis
Source: Eur Rev Aging Phys Act. 2025 May 1;22:6. doi: 10.1186/s11556-025-00372-z (PMC12044818; doi:10.1186/s11556-025-00372-z)
Supplement: Supplementary file 1 — Supplementary Material 1 [file 11556_2025_372_MOESM1_ESM.docx]

**Additional file**

| **Middle-aged women (n=1 158)** | 1. | 2. | 3. | 4. | 5. | 6. |
| --- | --- | --- | --- | --- | --- | --- |
| 1. Physical activity [mg] |  |  |  |  |  |  |
| 2. Total body mass [kg] | -.180 (.036) *** |  |  |  |  |  |
| 3. Muscle-free mass [kg] | -.226 (.035) *** | .970 (.007) *** |  |  |  |  |
| 4. Muscle mass [kg] | .025 (.037) | .694 (.022) *** | .497 (.028) *** |  |  |  |
| 5. Relative muscle mass [%] | .282 (.034) *** | -.654 (.023) *** | -.815 (.017) *** | .078 (.031) *** |  |  |
| 6. Six-minute walking distance [m] | .272 (.035) *** | -.301 (.032) *** | -.399 (.031) *** | .107 (.033) ** | .526 (.029) *** |  |
| 7. Relative knee extension torque [Nm/kg] | .213 (.037) *** | -.367 (.031) *** | -.461 (.030) *** | .053 (.035) | .571 (.027) *** | .490 (.030) *** |
| **Older women (n=289)** |  |  |  |  |  |  |
| 1. Physical activity [mg] |  |  |  |  |  |  |
| 2. Total body mass [kg] | -.310 (.057) *** |  |  |  |  |  |
| 3. Muscle-free mass [kg] | -.363 (.055) *** | .982 (.011) *** |  |  |  |  |
| 4. Muscle mass [kg] | .014 (.060) | .687 (.043) *** | .536 (.050) *** |  |  |  |
| 5. Relative muscle mass [%] | .425 (.054) *** | -.709 (.042) *** | -.824 (.034) *** | .005 (.060) |  |  |
| 6. Six-minute walking distance [m] | .511 (.051) *** | -.318 (.059) *** | -.373 (.058) *** | .017 (.061) | .441 (.055) *** |  |
| 7. Relative knee extension torque [Nm/kg] | .464 (.053) *** | -.370 (.055) *** | -.442 (.053) *** | .047 (.059) | .560 (.050) *** | .558 (.050) *** |
| **Older men (n=196)** |  |  |  |  |  |  |
| 1. Physical activity [mg] |  |  |  |  |  |  |
| 2. Total body mass [kg] | -.174 (.073) *** |  |  |  |  |  |
| 3. Muscle-free mass [kg] | -.285 (.071) *** | .954 (.022) *** |  |  |  |  |
| 4. Muscle mass [kg] | .154 (.072) * | .715 (.052) *** | .473 (.067) *** |  |  |  |
| 5. Relative muscle mass [%] | .423 (.070) *** | -.543 (.064) *** | -.765 (.048) *** | .187 (.077) * |  |  |
| 6. Six-minute walking distance [m] | .527 (.062) *** | -.196 (.074) ** | -.329 (.072) *** | .189 (.075) * | .491 (.070) *** |  |
| 7. Relative knee extension torque [Nm/kg] | .346 (.068) *** | -.283 (.070) *** | -.406 (.067) *** | .116 (.073) | .535 (.064) *** | .442 (.067) *** |

**Additional Table 1.** Bivariate correlations between the measures of physical activity, body composition, and functional capacity.

Multiple imputation was applied in the analyses. Data are Pearson correlation coefficient (standard error). *** p<0.001, ** p<0.01, * p<0.05.

**Additional Table 2.** Full structural equation model for middle-aged women (n=1 158).

|  | β | 95% CI | p-value |
| --- | --- | --- | --- |
| **Factor loadings for functional capacity** |  |  |  |
| Six-minute walking distance | 0.699 | (0.657, 0.741) | <0.001 |
| Relative knee extension torque | 0.706 | (0.644, 0.748) | <0.001 |
| **Regression paths to functional capacity** |  |  |  |
| Skeletal muscle mass | 0.566 | (0.484, 0.648) | <0.001 |
| Muscle-free mass | -0.899 | (-0.967, -0.831) | <0.001 |
| Age | -0.129 | (-0.199, -0.059) | <0.001 |
| Menopausal status: Peri | 0.029 | (-0.053, 0.110) | 0.489 |
| Menopausal status: Post | 0.065 | (-0.025, 0.154) | 0.158 |
| Work includes physical activity | -0.097 | (-0.166, -0.028) | 0.006 |
| **Regression paths to physical activity** |  |  |  |
| Functional capacity | 0.410 | (0.207, 0.613) | <0.001 |
| Skeletal muscle mass | -0.053 | (-0.193, 0.087) | 0.460 |
| Muscle-free mass | 0.058 | (-0.151, 0.268) | 0.584 |
| Age | 0.033 | (-0.048, 0.114) | 0.426 |
| Menopausal status: Peri | -0.001 | (-0.085, 0.084) | 0.987 |
| Menopausal status: Post | -0.007 | (-0.098, 0.083) | 0.874 |
| Work includes physical activity | 0.247 | (0.173, 0.321) | <0.001 |
| **Correlations with muscle-free mass** |  |  |  |
| Skeletal muscle mass | 0.506 | (0.457, 0.554) | <0.001 |
| Age | 0.039 | (-0.021, 0.100) | 0.200 |
| Menopausal status: Peri | 0.039 | (-0.022, 0.099) | 0.209 |
| Menopausal status: Post | -0.037 | (-0.101, 0.026) | 0.251 |
| Work includes physical activity | -0.038 | (-0.103, 0.028) | 0.264 |
| **Correlations with skeletal muscle mass** |  |  |  |
| Age | -0.085 | (-0.147, -0.023) | 0.007 |
| Menopausal status: Peri | 0.003 | (-0.058, 0.064) | 0.915 |
| Menopausal status: Post | -0.128 | (-0.188, -0.069) | <0.001 |
| Work includes physical activity | 0.006 | (-0.059, 0.071) | 0.852 |
| **Correlations with menopausal status: Peri** |  |  |  |
| Age | -0.070 | (-0.126, -0.014) | 0.014 |
| Menopausal status: Post | -0.572 | (-0.604, -0.540) | <0.001 |
| Work includes physical activity | 0.031 | (-0.031, 0.093) | 0.326 |
| **Correlation with menopausal status: Post** |  |  |  |
| Age | 0.359 | (0.307, 0.411) | <0.001 |
| Work includes physical activity | 0.092 | (0.030, 0.154) | 0.003 |
| **Correlation with work includes physical activity** |  |  |  |
| Age | 0.034 | (-0.028, 0.096) | 0.280 |

Reference group for menopausal status is premenopausal. Reference group for occupational physical activity is sedentary work; β, standardized path coefficient; CI, confidence interval.

**Additional Table 3.** Full structural equation model for older women (n=289).

|  | Path coefficient | 95% CI | p-value |
| --- | --- | --- | --- |
| **Factor loadings for functional capacity** |  |  |  |
| Six-minute walking distance | 0.758 | (0.697, 0.820) | <0.001 |
| Relative knee extension torque | 0.759 | (0.710, 0.809) | <0.001 |
| **Regression paths to functional capacity** |  |  |  |
| Skeletal muscle mass | 0.375 | (0.227, 0.523) | <0.001 |
| Muscle-free mass | -0.752 | (-0.885, -0.618) | <0.001 |
| Age | -0.321 | (-0.445, -0.198) | <0.001 |
| **Regression paths to physical activity** |  |  |  |
| Functional capacity | 0.696 | (0.497, 0.895) | <0.001 |
| Skeletal muscle mass | -0.001 | (-0.142, 0.140) | 0.985 |
| Muscle-free mass | 0.020 | (-0.183, 0.222) | 0.849 |
| Age | 0.089 | (-0.044, 0.221) | 0.189 |
| **Correlations with muscle-free mass** |  |  |  |
| Skeletal muscle mass | 0.541 | (0.412, 0.669) | <0.001 |
| Age | -0.008 | (-0.117, 0.102) | 0.890 |
| **Correlations with skeletal muscle mass** |  |  |  |
| Age | -0.233 | (-0.336, -0.130) | <0.001 |

β, standardized path coefficient; CI, confidence interval.

**Additional Table 4.** Full structural equation model for older men (n=196).

|  | Path coefficient | 95% CI | p-value |
| --- | --- | --- | --- |
| **Factor loadings for functional capacity** |  |  |  |
| Six-minute walking distance | 0.686 | (0.581, 0.792) | <0.001 |
| Relative knee extension torque | 0.673 | (0.584, 0.762) | <0.001 |
| **Regression paths to functional capacity** |  |  |  |
| Skeletal muscle mass | 0.572 | (0.366, 0.777) | <0.001 |
| Muscle-free mass | -0.821 | (-1.015, -0.628) | <0.001 |
| Age | -0.197 | (-0.362, -0.032) | 0.019 |
| **Regression paths to physical activity** |  |  |  |
| Functional capacity | 0.924 | (0.400, 1.448) | <0.001 |
| Skeletal muscle mass | -0.137 | (-0.530, 0.256) | 0.495 |
| Muscle-free mass | 0.293 | (-0.250, 0.836) | 0.291 |
| Age | 0.167 | (-0.023, 0.356) | 0.084 |
| **Correlations with muscle-free mass** |  |  |  |
| Skeletal muscle mass | 0.477 | (0.333, 0.621) | <0.001 |
| Age | 0.000 | (-0.147, 0.146) | 0.998 |
| **Correlations with skeletal muscle mass** |  |  |  |
| Age | -0.354 | (-0.477, -0.232) | <0.001 |

β, standardized path coefficient; CI, confidence interval.
